# Effect of health insurance program for the poor on out-of-pocket inpatient care cost in India: evidence from a nationally representative cross-sectional survey

**DOI:** 10.1186/s12913-020-05692-7

**Published:** 2020-09-07

**Authors:** Shyamkumar Sriram, M. Mahmud Khan

**Affiliations:** Department of Health Services Policy and Management, University of South Carolina, Columbia, SC USA

**Keywords:** Health insurance, Financial protection, Out-of-pocket health expenditure, Inpatient

## Abstract

**Background:**

In India, Out-of-pocket expenses accounts for about 62.6% of total health expenditure - one of the highest in the world. Lack of health insurance coverage and inadequate coverage are important reasons for high out-of-pocket health expenditures. There are many Public Health Insurance Programs offered by the Government that cover the cost of hospitalization for the people below poverty line (BPL), but their coverage is still not complete. The objective of this research is to examine the effect of Public Health Insurance Programs for the Poor on hospitalizations and inpatient Out-of-Pocket costs.

**Methods:**

Data from the recent national survey by the National Sample Survey Organization, Social Consumption in Health 2014 are used. Propensity score matching was used to identify comparable non-enrolled individuals for individuals enrolled in health insurance programs. Binary logistic regression model, Tobit model, and a Two-part model were used to study the effects of enrolment under Public Health Insurance Programs for the Poor on the incidence of hospitalizations, length of hospitalization, and Out-of- Pocket payments for inpatient care.

**Results:**

There were 64,270 BPL people in the sample. Individuals enrolled in health insurance for the poor have 1.21 higher odds of incidence of hospitalization compared to matched poor individuals without the health insurance coverage. Enrollment under the poor people health insurance program did not have any effect on length of hospitalization and inpatient Out-of-Pocket health expenditures. Logistic regression model showed that chronic illness, household size, and age of the individual had significant effects on hospitalization incidence. Tobit model results showed that individuals who had chronic illnesses and belonging to other backward social group had significant effects on hospital length of stay. Tobit model showed that days of hospital stay, education and age of patient, using a private hospital for treatment, admission in a paying ward, and having some specific comorbidities had significant positive effect on out-of-pocket costs.

**Conclusions:**

Enrolment in the public health insurance programs for the poor increased the utilization of inpatient health care. Health insurance coverage should be expanded to cover outpatient services to discourage overutilization of inpatient services. To reduce out-of-pocket costs, insurance needs to cover all family members rather than restricting coverage to a specific maximum defined.

## Background

Achieving Universal Health Coverage (UHC) is an important goal for almost every nation in the world [1]. Financial risk protection is one aspect or dimension of UHC and providing financial risk protection is a specific target of the Sustainable Development Goals (SDGs) of the United Nations [2]. The level of financial protection realized by different population groups depends on the out-of-pocket expenditures (OOP) incurred by them for financing health care [3, 4]. High OOP health expenditures, by definition, happens when households decide to access and utilize health care services but do not have protection against high expenditures due to high medical care costs and/or lack of access to insurance coverage and other safeguards against out of pocket costs [5]. Evidence from Indian National Health Account 2017 shows that OOP health expenditures for inpatient care constitutes around 32% of the total OOP health expenditures, despite the coverage offered by various health insurance programs [6]. The public healthcare system in India, with geographically distributed primary health centers and sub-centers, is very weak and lacks basic infrastructure. In addition, waiting times in the public sector primary health care facilities are very long, encouraging most of the patients to choose private providers for their health care needs [7–9]. Increasing propensity to use private sector health care providers increases the costs and lack of health insurance coverage and inadequate coverage make the OOP expenditure high with negative impacts on health care utilization [10]. Since cost of inpatient services is high, protecting households from hospital OOP expenses should significantly improve financial equity in health service delivery. Moreover, access to health care can be improved significantly if the system can protect the poor households from significant OOP expenses. In order to improve access to health care by the poor, India initiated a number of health insurance programs since 2008 [10]. This paper advances our knowledge about financial risk protection and effect of health insurance programs for the poor on access, utilization and out-of-pocket expenses in India.

The increase in health insurance coverage may lead to increase in health care utilization because of the change in behavior of the insured as well as the health care provider. A study by Anderson et al. (2012) in the USA found that there was a 61% reduction in inpatient hospital admissions and 40% reduction in emergency department visits among the uninsured population compared to insured population with similar sociodemographic characteristics [11]. Evidence from literature has shown that increased health insurance coverage leads to increase in utilization of health services, but the effect of health insurance coverage on financial risk protection is less clear, especially for poor beneficiaries [12]. This is because, there are two opposing forces in play due to increased coverage of insurance; one aspect is the increased access and utilization due to insurance coverage, which increases total health care cost and second, even with lower OOP rates per service, total OOP may actually become higher due to higher utilization. The health insurance for the poor in India covers only inpatient services. This creates an incentive for the patients to visit hospitals and get hospitalized, instead of using basic primary health care services. Studies on hospitalization trends in India showed that an annual hospitalization rate increased from 16.6 per 1000 population to 37.0 per 1000 from 1995 to 2014 [13]. Although, we expect to see an increase in hospital utilization rate with improving access and availability, a part of this increase may be due to hospital insurance offered to the poor by the Government of India.

There are many Public Health Insurance Programs for the Poor offered by the Government of India (GOI) and some states to cover the cost of hospitalization and inpatient care [14]. RSBY is a health insurance program started by the Ministry of Labor and Employment of the GOI in April 2008 and it provides a wide range of hospital-based healthcare services to Below Poverty Line (BPL) families [15]. There are a number of state-run public health insurance programs for the poor in three of the southern states in India which provide higher coverage than RSBY and are exempted from the national program. The programs are the Chief Minister’s Comprehensive Health Insurance Scheme in Tamil Nadu State, Rajiv Aarogyasri Community Health Insurance (RACHI) in Andhra Pradesh State, and Vajpayee Aarogyasri Scheme (VAS) in Karnataka State [14]. Table 1 summarizes the important features of the national RSBY program and the state health insurance programs for the poor in the states of Andhra Pradesh, Karnataka, and Tamil Nadu.

**Table 1:** Key Parameters under Health Insurance Programs in India

Parameter	Rashtriya Swasthiya Bima Yojana (RSBY)		State health insurance programs for the poor (Andhra Pradesh, Tamil Nadu and Karnataka)
	Description	Additional Caveats	Description
Benefits covered	Cost of hospitalization for 725+ procedures at empaneled hospitals up to INR 30,000 per annum per household; INR 100 per visit up to INR 1000 per year for transport cost	Pre-existing conditions are covered; minimal exclusions; day surgeries covered; outpatient expenditure is not covered	Andhra Pradesh - Families are provided coverage for INR 200,000 per family per year, and there are no restrictions on the number of family members enrolled Karnataka - INR150,000 per year for 5 persons in a family Tamil Nadu –INR100,000 per family per year
Eligibility criteria	Must be on the official state BPL list; Limited to five members of the household including household head, spouse, and three dependents	All enrolled members must be present to be enrolled;	Must be on the official BPL list of the specific state. No restrictions on the number of family members enrolled in Andhra Pradesh, and Tamil Nadu. Covers five members of family in Karnataka.
Premium and fees	INR 30 registration fee per household per annum paid by household.		No specific enrolment fee in the three states of Andhra Pradesh, Karnataka, and Tamil Nadu
Financing	75%/ 25% Government of India/ State Government	The ratio is 90% /10% in Northeast states and Jammu & Kashmir	Completely funded by the respective states
Insurer	Both public and private insurance companies can bid to work in a district or more than a district recommended by state governments	In one district only one insurance company is finally selected	Both public and private insurance companies can bid to work at the state level
Service provider	Both public and private sector service providers can apply to join the network of providers empaneled under the scheme	Minimum eligibility criteria on quality of services to be provided have been laid down by the MoL & E	Both public and private sector service providers in the specific state can join the network of providers empaneled in the program. Minimum eligibility criteria laid down by the respective State Health Ministries

Source: Ministry of Labor and Employment (MoL & E) and State Health Departments

Around 41 million families are enrolled in RSBY, covering around 150 million poor people as of September 2016. The enrolment under the program has been increasing starting from only 55 districts in 2008–2009. Nationally, around 460 districts participate in the program, with 57% of the eligible households are currently enrolled [16]. There is significant inter-district and inter-state variation in the percentage of eligible households enrolled in RSBY. Across states, the degree of enrolment of households varies from a low of 24% in Arunachal Pradesh and 36% in Haryana to more than 75% in Kerala. The degree of enrollment of households by district varies significantly across the country, with a low rate of enrollment of 3% in Kannauj district and 6% in Kanpur district in the Uttar Pradesh state to a high enrollment rate of 90% of households in most of districts in the Chhattisgarh and Kerala states of India. Enrolment is not complete in many states, even a decade after the start of the program. Also, as of September 2016, the state of Rajasthan was still in its early stage for enrolling households in RSBY [16]. This shows that enrollment in the RSBY program has been slow in some parts of India. Not all states in India participate in RSBY. The state of Andhra Pradesh has not adopted RSBY as it already has a substantially more generous state level health insurance program than RSBY which pre-dates RSBY with relatively high population coverage, covering nearly 80% of its population [17]. Studies show that coverage rate of RSBY is low with half of the poor individuals not covered because of problems with targeting due to incomplete information on poor individuals and households, high migration rates among the poor [16] and possibly the rapid changes in social mobility.

Under the Public Health Insurance Programs for the poor only the hospitalization services and expenses associated with inpatient care are covered. It is expected that the health insurance for the poor will increase utilization of hospital services by the BPL households who would usually be forced to postpone their non-urgent procedures for a later time because of cost. Even with insurance, there may be OOP payments for drugs, tests and post-treatment care which are not covered by the health insurance. Therefore, hospital insurance may actually end-up increasing the OOP payments for inpatient and inpatient-related care for the poor. Hence the direction of effect of the Poor People Health Insurance Programs on total inpatient OOP health expenditure is unclear. Also, RSBY may lead to misuse of services, since both the physician and the patient have the incentive to convert an outpatient case into an inpatient admission, leading to unnecessary utilization [18]. The objective of this research is to examine the effect of Public Health Insurance Programs for the Poor on incidence of hospitalizations and inpatient OOP health expenditures.

Many studies show that people incur high OOP health expenditures despite being covered by the national health insurance program RSBY or other state health insurance programs [19–24]. However, studies on state health insurance programs in Karnataka and Andhra Pradesh showed that OOP health expenditures significantly declined with health insurance coverage [17, 25, 26]. Cross-sectional studies done in Tamil Nadu and Maharashtra show that the utilization of healthcare was significantly higher among the insured compared to the uninsured population [27].

Previous studies on Poor People’s Health Insurance Programs such as RSBY dealt with issues related to program enrolment [28], barriers in implementation of the program [22], effect of information campaign [29], hospitalization patterns [30], and determinants of participation in the program [31]. There are only two district level studies on RSBY, one done in Amaravati district in Maharashtra [32] and the other in Gujarat [19], that showed increased hospitalizations and higher OOP health expenditures among the RSBY insured individuals. The study in Gujarat found that RSBY enrollees experienced higher OOP health expenditures because they had to pay for medicines and diagnostics during the hospital admission [25]. In contrast, another state level study for the Aarogyasri program found that insurance significantly reduced the OOP health expenditures for hospitalizations [17]. Most of other studies that studied the effect of health insurance on hospitalizations and OOP health expenditures were community-based health insurance programs in different parts of the country [25, 33–35] and thus limiting its usefulness for national decision-making.

This study is a considerable improvement over other studies on Public Health Insurance Programs for the Poor in India on two important counts: i) the study uses nationally representative dataset which helps in estimating pan-India effects of Public Health Insurance Programs for the Poor ii) the study evaluates the effect of Public Health Insurance Programs for the Poor by comparing outcomes between poor people enrolled and not-enrolled in the insurance program. Many studies are based on RSBY enrollees alone and do not have any controls making it difficult to identify the effects of the Public Health Insurance Programs for the Poor. This study identified comparable control population from among those who are poor but were not enrolled in the insurance program. The specific research questions that will be addressed in this research are: (i) How do hospitalizations differ between the enrolled and not-enrolled groups under Public Health Insurance Programs for the Poor? and (ii) How does OOP health expenditure for inpatient care differ among people enrolled and not-enrolled under Public Health Insurance Programs for the Poor?

## Methods

### Data source

The data from the National Sample Survey Organization (NSSO) of the GOI were used for the study [36]. The NSSO is a national organization under the Ministry of Statistics and Implementation which was established in 1950 to regularly conduct surveys and provide useful statistics in the field of socio-economic status of households, demography, health, industries, agriculture, consumer expenditure etc. The specific data set from NSSO that was used in this study is the Social Consumption (Health), NSS 71st Round for 2014, which is the latest nationwide data available for India. The survey covered whole of the Indian Union. The survey used the interview method of data collection from a sample of 65,932 randomly selected households (36,480 in rural India and 29,452 in urban India) and 335,499 individuals, covering the members of the household in all the 36 states (including union territories). The data for the survey were collected over a period of six months, from January to June 2014. The NSSO Social Consumption (Health) collected data on demographic characters, employment, health conditions, source of payments, health insurance coverage, type of coverage, costs of various inpatient services, level of care, type of care and a number of other variables. The survey also collected information on medical care received at inpatient and outpatient facilities of medical institutions including health expenditures for various episodes of illness. This is the first NSSO health survey that collected data on utilization of alternative medicines. The details of hospitalization for all current and former members of the household were collected for the last 365 days (hospitalization occurred from January 2013 to June 2014) and the details of outpatient services were collected for the last 15 days.

### Estimation of OOP health expenditures

‘Total Out-of-Pocket health expenditures for inpatient care’ is defined as the total health expenditure for inpatient care net of reimbursement by health insurance. It is a continuous variable calculated in Indian Rupees (INR). In the data provided by the government of India, hospitalization expenses were included under two heads namely medical (direct) and direct non-medical (indirect) costs. Direct medical expenditure consists of package component and non-package component (doctor fee, medicines, diagnostic tests, bed charges, other medical expenses) and direct non-medical expenditure consists of transport for patient, transport for others, lodging charges of escort, food expenses, and other expenses. There is a separate variable in the data which provided the “amount reimbursed by the health insurance”. All these variables were used to derive the OOP health expenditure for inpatient care.

Total inpatient healthcare expenditure = (Medical expenditure, X) + (Direct Non-Medical.

Expenditure, Y).

Total out-of-pocket inpatient health expenditure = (Total inpatient healthcare expenditure) –.

(Amount reimbursed by the health insurance, Z)

$$ T=\left(X+Y\right)-Z $$

### Empirical methodology

The main objective of this study is to estimate the effect of Public Health Insurance Programs for the Poor on hospitalizations and OOP inpatient care costs. The effects of the program were estimated by comparing the probability of hospitalizations and OOP inpatient healthcare costs between the groups who are eligible (poor) and covered by the insurance programs and who are eligible (poor) but not covered. In theory, the best approach of estimating the impact of a program is to adopt a Difference-in-difference (DID) framework with randomized allocation of eligible individuals in the program group and the no-program group. The framework requires data on the two groups in the pre-intervention period and then in the post-intervention period [37]. DID estimators compare the change in mean outcomes before and after the intervention among individuals who acquire coverage (treated) and those remaining not exposed.

To estimate the causal effect using DID, the assumptions of DID must be satisfied. The main assumptions are that the treatment and control groups have parallel trends in outcome, the composition of the treatment and control groups are stable for repeated cross-sectional design, the allocation of treatment is unrelated to the outcome at baseline, and there are no spillover effects. The most important assumption for DID is the ‘parallel trend assumption’. This means that in the absence of the intervention/treatment, the average difference in the outcome between the treatment and control groups would have remained constant in post-intervention time period as in pre-intervention period. The violation of this assumption will imply that the DID approach will not be able to obtain unbiased estimates of program impacts. The DID model cannot be used if composition of the pre-intervention and post-intervention groups are not stable, if the comparison group has a different outcome trend, and if the allocation of the treatment/intervention is determined by the baseline outcome [37].

However, the treated and untreated may differ in the distribution of both observable and unobservable characteristics. Heckman and Vytlacil (2007) highlighted that unobservable variables may play a bigger (or smaller) role in influencing the with-treatment outcome than the without-treatment outcome [38]. Inability to control for them is likely to provide under (over) estimation of the effects of the programs. Since the main assumption of DID is parallel trend assumption and checking for the constant difference in outcome over time is necessary for deriving impact of a program or intervention using DID approach.

For the purpose of this study, a number of simplifying assumptions must be made as the data set is cross-sectional in nature and we only observe the outcomes in the year the data were collected. Therefore, the data set does not provide any information on the individuals who were enrolled in the insurance program in the previous period and those who were not enrolled. The insurance program is designed for the poor households and since belonging to the poverty group is a dynamic event, a household in poverty in pre-insurance period may not necessarily be in poverty in the post-intervention period. Moreover, household in poverty in the current year (the year of data collection) may not have been in poverty in the previous period. Almost all programs also show some degree of mistargeting implying that some poor people may not be offered the insurance while some non-poors are offered the insurance benefit. These potential deviations from expected enrollment may affect the estimate of outcomes when a post-intervention year’s data are used.

**Fig. 1:**
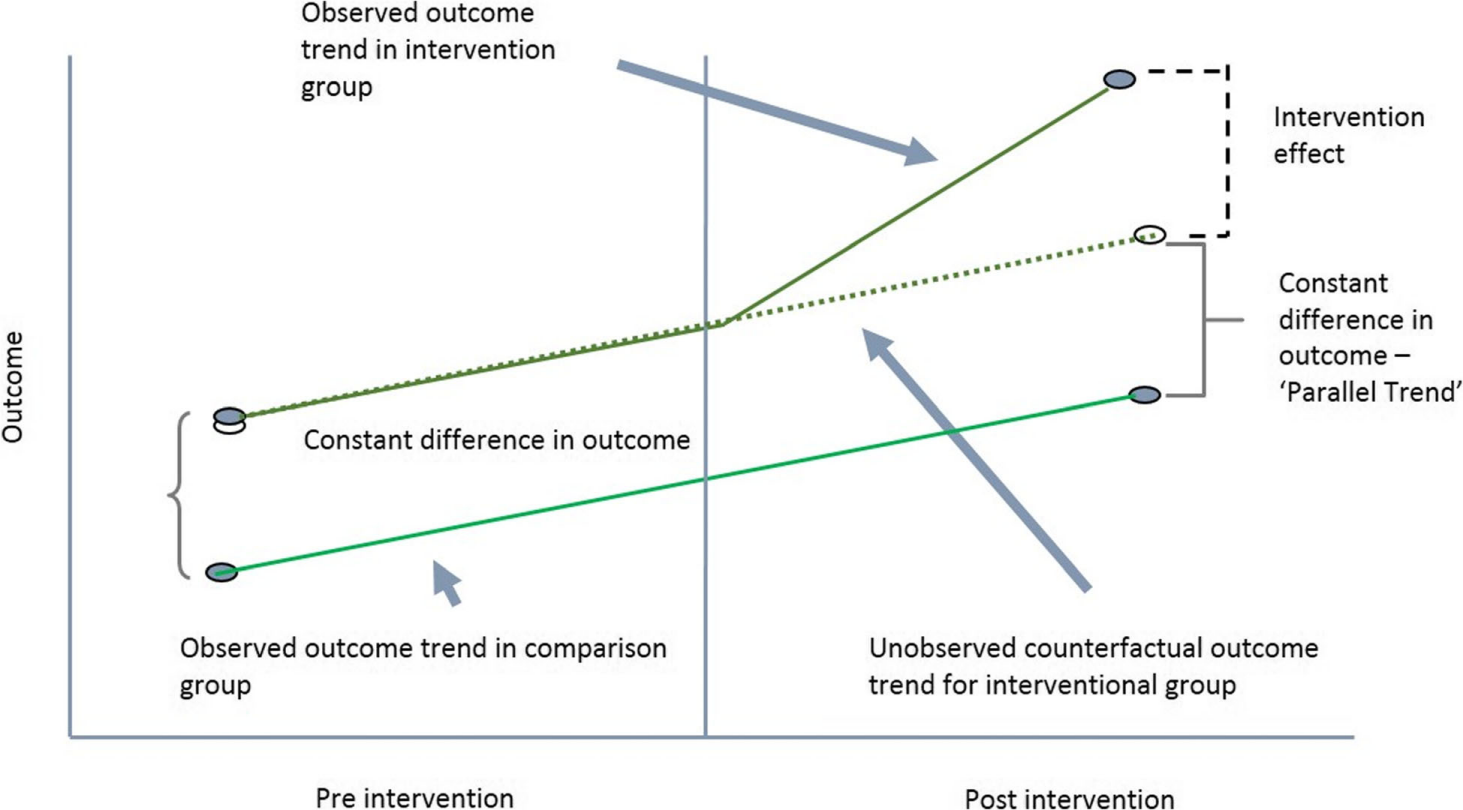
Intervention Effect using Difference-in-Difference Method

In the DID model, the intervention effect will be the difference between the observed outcome in intervention group and the unobserved counterfactual outcome for intervention group as shown in Fig. 1. It is possible to model the unobserved counterfactual outcome for intervention group in the post-intervention period in absence of the intervention if data on pre-intervention period are available. In the cross-sectional data of the study, we do not have information on the intervention and control groups in pre-intervention period and if intervention and control groups differed in terms of outcomes of interests, we have no way of correcting for this. The only alternative approach we can adopt is to select the comparison groups from the cross-sectional data in such a way that the likelihood of pre-intervention variability would be minimized.

**Fig. 2:**
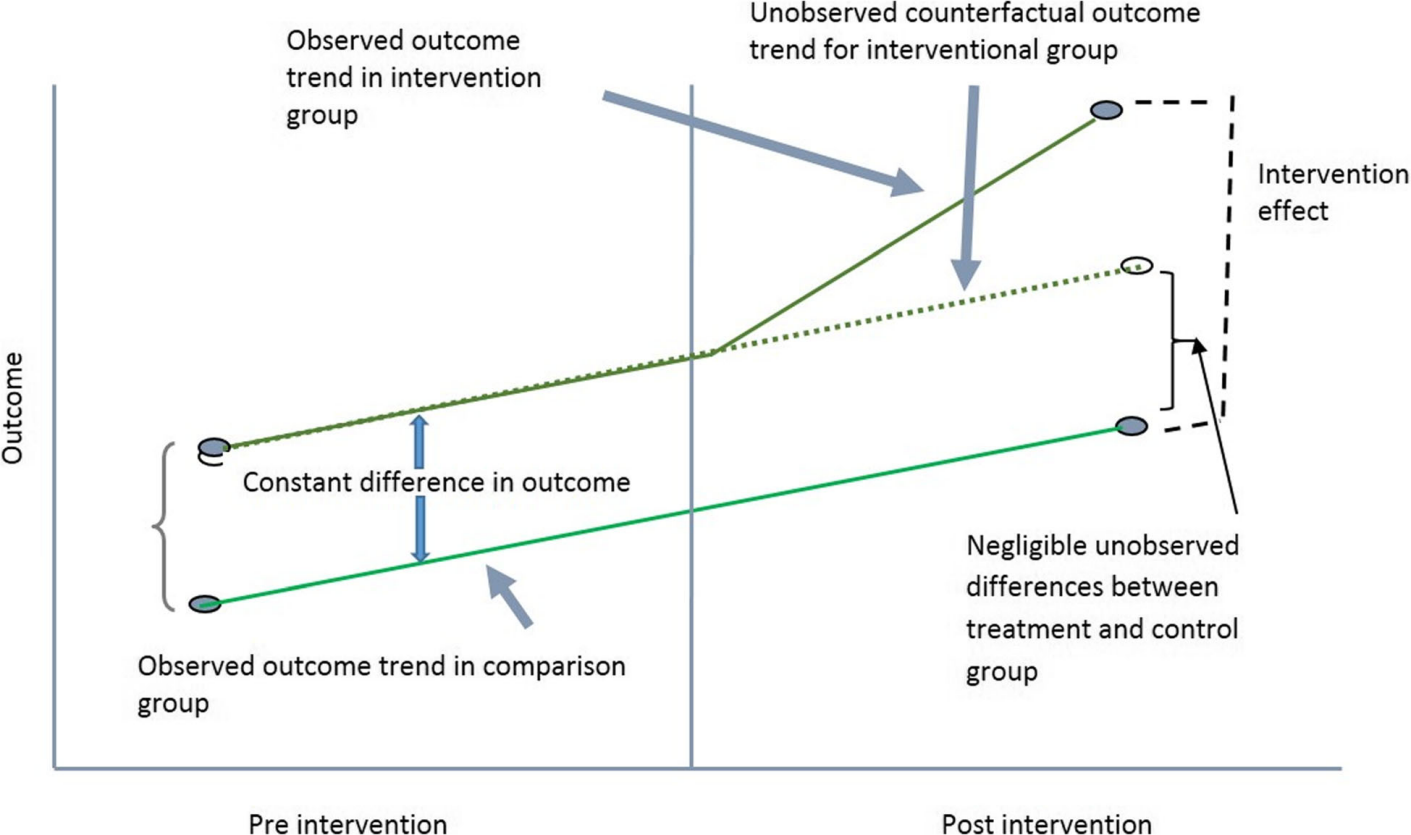
Intervention Effect using Cross-sectional data

Rather than identifying the economic status of individuals who were actually covered by insurance in the previous period, the implicit assumption we are using is complete absence of mistargeting or simply not allowing the mistargeted individuals to be in the analysis. It is also assumed the social mobility of poor households in India is relatively low and so the households belonging to poverty category in the current year (the year of the survey) were also poor in the previous few years. If the enrolment in the program by the poor is completely random, the unobserved characteristics will become increasingly similar between program participants and non-participants with increasing sample size. In the data set, the sample size of BPL individuals is quite large but, clearly, enrolment in programs are almost never purely random. For the insurance program in India, it is not clear how exactly the households were selected for enrollment, especially because so many of the poor households were not in the program. It appears that administrative listing of poor households rather than self-selection guided the enrolment. This process, to some extent, can reduce the variability between enrolled and non-enrolled poors in terms of both observable and unobservable variables. Although the observable differences can be controlled for, it is not possible to ensure minimization of unobserved differences between the groups. In the analysis, it is assumed that the factors other than insurance coverage that may cause differences between the intervention group and control group in terms of utilization of hospital services or out-of-pocket costs would be relatively low. If the intervention and control groups are matched using a list of observable characteristics, it further reduces the possibility of biased estimate or unequal starting points in terms of outcome variables. Thus, using the cross-sectional post-intervention data, the intervention effect will be the difference between the observed outcome in the intervention group and the observed outcome in the control group as shown in Fig. 2.

Two important assumptions are made in the impact evaluation process when using this cross-sectional data. The assumptions are, at the starting point in the pre-intervention period, the unobservable differences between the intervention and control group are small, if any, and that both the intervention group and the matched control group would show similar trend in terms of outcomes in absence of the intervention.

### Treatment group, control group and propensity score matching

The treatment group consist of all the poor people currently enrolled under the Public Health Insurance Programs for the Poor namely the RSBY and other state health insurance programs for the poor. The control group will consist of all people who are poor but not enrolled in the Public Health Insurance Programs for the Poor. In order to make both the groups comparable and to avoid selection bias, a propensity score matching was used to match the treatment and control groups. A propensity score is the conditional probability that a subject receives “treatment” given the subject’s observed covariates. A propensity score matched regression analysis incorporating survey weights can better account for selection bias based on observed variables than an unmatched regression [39, 40]. The main goal of propensity score is to balance the observed covariates from the individuals in the treatment and control groups in order to imitate a randomized study [41]. The variables used to get the propensity scores were education, socioeconomic status, location of household (urban/rural), household size, and age of the individual, using a user-written command *psmatch2* in STATA. After matching, a regression analysis was performed.

### Data analysis

#### Incidence of hospitalization and length of hospital stay

To study the effects of enrolment under Public Health Insurance Programs for the Poor on the incidence of hospitalizations after controlling for other factors, a binary logistic regression model was used. The logistic regression model is preferred since the dependent variable is dichotomous. “Whether the individual was hospitalized during the last 365 days?” was used as the dependent variable. A dichotomous variable for hospitalization was created with 0 for ‘not hospitalized during the last 365 days’ and 1 for ‘hospitalized during the last 365 days’. The independent variables include enrollment under the Poor People Health Insurance Program and other covariates. The model estimated the log odds of incidence of hospitalization adjusted for a set of explanatory variables. Individual is the unit of analysis. The results for the logistic regression are shown with the estimated regression coefficients, odds ratios and 95% confidence intervals. Tobit Regression Model was used to study the association between the Public Health Insurance Programs for the Poor and the length of stay in hospitals. The Tobit model is usually estimated when the dependent variable has a large number of observations clustered around a specific value, usually around zero. For the length of hospital stay, the dependent variable is either zero or higher than 0 [42]. Length of stay, by definition, is truncated below zero and thus the Tobit model is used.

#### OOP inpatient healthcare cost

Tobit Regression Model has also been used to study the association between Public Health Insurance Programs for the Poor and the OOP cost for inpatient care. Similar to length of stay, OOP cost is always positive or zero with a relatively high proportion showing zero OOP expenses.

The empirical equation for the Tobit model can be written as:

$$ {Y}_i^{\ast }={\beta}_0+{\beta}_1{X}_1+{\beta}_2{X}_2+\dots +{\beta}_k{X}_k+\mu $$

$$ {Y}_i={Y}_i^{\ast }\  if\ {Y}_i^{\ast }>0 $$

$$ {Y}_i=0\  if\ {Y}_i^{\ast }>0 $$

where ${Y}_i^{\ast }$ is the latent dependent variable, and *Y*_*i*_ is the observed values of the dependent variable.

## Results

### Descriptive statistics

The total sample consisted of 336,470 individuals. In the total sample, 42,121 individuals were covered by the government sponsored health insurance programs such as Employee’s State Insurance Scheme (ESIS), Central Government Health Scheme (CGHS), and the poor people’s health insurance programs such as RSBY and other state health insurance programs. Poverty is a dynamic event where people move in and out of poverty. We used the poverty line for 2014 to identify individuals who were poor in 2014. Since the data had only one variable for the individuals covered by the government sponsored health insurance programs which included both the poor people health insurance programs and other government health insurance programs for the non-poor, we assumed that the people who were below the poverty line and enrolled in the government sponsored health insurance programs are enrolled in the public health insurance programs for the poor such as RSBY, RACHI etc. Since eligibility in the insurance programs like RSBY was defined by poverty status alone, the poor individuals not enrolled in the program were clearly eligible but not covered. Only the poor people below the poverty line as of 2014 has been used for defining eligibility for the public insurance in this study.

Descriptive statistics for categorical variables are presented in Table 2 for the poor individuals in the sample. The survey collected data from 64,270 poor individuals. Only 9.55% of the poor individuals in India were enrolled in any type of public health insurance programs for the poor. About 9.41% of the poor individuals were enrolled in RSBY in all states of India excepting Tamil Nadu, Andhra Pradesh, and Karnataka. In Andhra Pradesh, 40% of the poor people were enrolled in RACHI, 5.7% were enrolled in VAS in Karnataka, and only 4.5% were enrolled in CCHIS in Tamil Nadu. Around 41.3% of the poor in the sample were illiterate; 80.6% belonged to Hindu religion; 85.1% were from the disadvantaged classes; 64.2% belonged to medium sized households (5 to 8 members). About 2.5% of the poor individuals were suffering from chronic illnesses.

**Table 2:** Descriptive Statistics for selected categorical variables for poor individuals in the 2014 survey of India

Variables	Categories	Frequency (%) ***n*** = 64,270	Weighted Percentage
Hospitalization (Yes/No)	Yes	7515 (11.69%)	3.33%
Health Insurance for the Poor	Enrolled	5917 (9.21%)	9.55%
Sex	Female	32,152 (50.03%)	48.90%
Marital Status	Never married	32,938 (51.25%)	51.81%
	Currently married	28,443 (44.26%)	43.59%
	Widowed/divorced/separated	2889 (4.50%)	4.60%
Education	Illiterate	26,063 (40.55%)	41.30%
	Primary/middle school	29,240 (45.50%)	47.39%
	Secondary school	4834 (7.52%)	6.49%
	Higher secondary school	2795 (4.35%)	3.46%
	Diploma/graduate/post graduate	1337 (2.08%)	1.36%
Location (Rural or Urban)	Rural	42,590 (66.27%)	80.03%
Religion	Hinduism	46,464 (72.30%)	80.57%
	Islam	11,836 (18.42%)	15.09%
	Christianity	3988 (6.21%)	2.09%
	Other religions	1982 (3.08%)	2.25%
Social Group	Scheduled tribes	12,983 (20.20%)	16.65%
	Scheduled castes	13,759 (21.41%)	25.51%
	Other backward classes	26,105 (40.62%)	42.97%
	Others	11,423 (17.77%)	14.86%
Household size	Small household (1–4 members)	8835 (13.75%)	18.07%
	Medium household (5–8 members)	39,009 (60.70%)	64.20%
	Large household (9 and more)	16,426 (25.56%)	17.73%
Household type	Self-employed	33,211 (51.67%	49.44%
	Regular wage/salary earning	7794 (12.13%)	9.27%
	Casual labor	21,617 (33.63%)	38.49%
	Others	1648 (2.56%)	2.80%
Latrine type	Service and pit latrine	13,594 (21.15%)	14.65%
	Septic tank/flush system	16,931 (26.34%)	19.36%
	No latrine and others	33,745 (52.51%)	65.99%
Drainage type	Open	30,535 (47.51%)	44.05%
	Covered	8543 (13.29%)	10.66%
	No drainage	25,192 (39.20%)	45.29%
Drinking water	Safe water	61,807 (96.17%)	98.36%
	Unsafe water	2463 (3.83%)	1.64%
Cooking fuel	Unclean fuels	50,913 (79.22%)	84.91%
	Clean fuels	12,802 (19.92%)	13.69%
	No cooking arrangement	555 (0.86%)	1.40%
Chronic illness	Yes	1911 (2.97%)	2.51%
Level of care (those who sought care from hospitals)	Sub-center/PHC/CHC	890 (1.38%)	0.42%
	Public hospital	4005 (6.23%)	1.72%
	Private hospital	2620 (4.08%)	1.18%
Type of ward (those who used hospitals)	Free	4532 (7.05%)	2.00%
	Paying general	2672 (4.16%)	1.20%
	Paying special	311 (0.48%)	0.13%
Ailment/disease or morbidity type for those seeking care	Infections	1518 (2.36%)	0.53%
	Cancers, blood, endocrine, metabolic, eye & ear diseases	486 (0.76%)	0.19%
	Cardiovascular, respiratory diseases	542 (0.84%)	0.22%
	Gastrointestinal diseases	553 (0.86%)	0.22%
	Skin, musculoskeletal, psychiatric & neurological diseases	576 (0.90%)	0.21%
	Genitourinary, obstetric & childbirth	3204 (4.99%)	1.73%
	Injuries	636 (0.99%)	0.23%
	Did not seek care	56,755 (88.31%)	96.67%

Table 3 reports the descriptive statistics for some continuous variables. The mean age of the poor population in the sample was 25.3 years. In terms of incidence of hospitalizations, only 3.3% of the weighted sample reported at least one hospitalization in the previous one year. The overall average days of hospital stay per poor person was only 0.17 days implying that average length of stay per admission as well as the proportion of poor individuals hospitalized in a year are quite small in general. The annual OOP health expenditure for inpatient health care for the whole poor population was 269.26 INR with average of annual consumption expenditure of 8505.62 INR. Therefore, on the average, poor households spent about 3% of their total annual consumption expenditure to inpatient care.

**Table 3:** Descriptive Statistics for continuous variables for poor individuals in the 2014 Survey

Variables		Weighted Mean	Standard Error	95% CI
Age (Years)		25.29	0.1719	24.95–25.63
Age by age-groups	0–18 years	9.21	0.0685	9.08–9.35
	19–40 years	29.41	0.1003	29.21–29.60
	41–60 years	50.06	0.1431	49.78–50.34
	61–80 years	67.71	0.2262	67.27–68.16
	81 years or higher	86.62	0.5686	85.50–87.74
Duration of hospitalization (days of stay per individual)		0.1664	0.0067	0.1532–0.1796
Annual inpatient OOP expenditure (INR) per individual		269.26	12.13	245.47–293.04
Annual consumption expenditure per individual (INR)		8505.62	18.5608	8269–8342

Table 4 shows the descriptive statistics for the poor individuals who were hospitalized during the recall period. Since the same person can be hospitalized more than once in a year, the length of hospital stay does not represent length of stay per admission. The mean duration of hospital stay was about five days for the poor individuals hospitalized in the year. The weighted average age of hospitalized individuals was 30.9 years in 2014. For this group, yearly consumption expenditure was 8449 INR and the yearly inpatient OOP health expenditure was 8149 INR. We mentioned earlier that the poor individuals, on the average, paid OOP about 3% of their annual consumption expenditure on inpatient care but if we focus on those who were actually hospitalized during the year, more than 95% of their annual consumption expenditure was due to OOP expenses related to inpatient care. Clearly, inpatient OOP expenditure has remained very high for the poor individuals in India.

**Table 4:** Descriptive Statistics of Variables for Poor Individuals who were Hospitalized at least once in the 365 days prior to the survey

Variable	Mean	Standard Error	95% Confidence Interval
Duration of hospitalization (days)	5.009	0.1605	4.686–5.315
Yearly Inpatient OOP health expenditure (INR)	8149.415	317.9662	7526.11–8772.71
Age (years)	30.927	0.3844	30.174–31.681
Yearly individual consumption expenditure (INR)	8449.035	46.2932	8358.287–8539.782

One to One propensity score matching was done using a STATA user-written command (*psmatch2*) by incorporating education, socioeconomic status, location of household (urban/rural), household size, and age of the individual as the matching variables. The results are reported in Table 5. Number of individuals in the intervention group, 5917 in total, were matched with 5917 in the control group. Thus, the total matched sample consisted of 11,834 observations. After matching, various empirical modelling were carried out using the total matched sample. Using the matched sample ensures that we are comparing similar poor individuals in both enrolled and non-enrolled groups.

**Table 5:** One to One Propensity Score Matching of poor individuals with insurance with poor individuals without the insurance coverage

	Treated	Control	Difference	T statistics	S. E
Total sample	5917	5917			
Average Treatment on Treated (ATT)	0.1407	0.1191	0.0216	2.89	0.0074
Propensity Score Testing of Two Groups					
	**Treated** **(Mean)**	**Control** **(Mean)**	**% Bias**	**T statistics**	**Probability(t)**
Age	26.821	26.426	2.0	1.10	0.269
Individual Consumption Expenditure	8588.9	8595.4	−0.3	−0.17	0.866
Household size	2.0255	2.014	1.9	1.04	0.299
Location	1.2505	1.2525	−0.4	−0.25	0.799
Education	1.7828	1.7725	1.2	0.67	0.503

### Multivariate analysis

The logistic regression model results for the effects of poor people health insurance program on incidence of hospitalization are shown in Table 6. People enrolled in poor people health insurance program have 1.23 higher odds of incidence of hospitalization compared to poor people without health insurance. Chronic illness, household size, and age of the individual show significant effects on incidence of hospitalization. Individuals with chronic illnesses have higher probability of hospitalization compared to individuals without any chronic conditions. All the age groups show higher probability of hospitalization compared to the reference age group of less than 18 years. Interestingly, individuals belonging to the medium and large size households had lower probability of incidence of hospitalization compared to individuals from small households. Social group, religion, urban/rural location, household type, marital status, education, number of hospital beds in the state were not significant in explaining variability in the incidence of hospitalizations. Fixed effects for state of residence of the individual was included in the model but no significant effects of the state of residence were found in the empirical analysis.

**Table 6:** Logistic Regression Results for the Effect of Poor People Health Insurance Program on the Incidence of Hospitalization

Incidence of Hospitalization	Odds Ratio	95% CI	***P*** value
Public Health Insurance for the Poor			
Not enrolled (Reference)			
Enrolled	1.23	1.06–1.44	0.007
Social Group			
Other Backward Classes (Reference)			
Scheduled tribes	1.01	0.85–1.19	0.878
Scheduled castes	1.01	0.86–1.19	0.859
Others	1.17	0.96–1.42	0.103
Chronic Illness			
No Chronic illness (Reference)			
Chronic Illness	3.55	2.87–4.45	< 0.001
Age Groups			
0 to 18 years (Reference)			
19 to 40 years	1.06	0.82–1.36	0.635
41 to 60 years	2.44	1.89–3.15	< 0.001
61 to 80 years	2.99	2.14–4.17	< 0.001
Older than 80 years	4.85	1.71–13.69	0.003
Interaction Age Group* Sex			
Female and Age Group (19 to 40 years)	6.81	4.95–9.36	< 0.001
Female and Age Group (41 to 60 years)	0.91	0.63–1.30	0.617
Female and Age Group (61 to 80 years)	0.82	0.51–1.30	0.411
Female and Older than 80 years	0.76	0.19–3.04	0.703
Household Size			
Small household (Reference)			
Medium household (5 to 8 members)	0.77	0.66–0.89	< 0.001
Large household (9 & more members)	0.47	0.39–0.58	< 0.001
Hospital beds per 1000 population			
More than 1 bed per 1000 (Reference)			
0.5 to 1 per 1000 population	1.59	0.34–7.40	0.551
Less than 0.5 per 1000 population	1.16	0.26–5.05	0.843
Constant	0.15	0.03–0.68	0.013

Table 7 presents the Tobit model results on the effect of poor people health insurance program on the duration or length of hospitalization. Being enrolled in health insurance for the poor had no significant effect on the duration of hospitalization. People who did not have chronic illnesses had significantly lower duration of hospitalization compared to people with chronic illnesses. People belonging to “other backward classes” category had significantly higher duration of hospitalization compared to the reference group (scheduled tribes). Other covariates such as household type, religion, age, urban/rural location, household size, marital status, education, and number of hospital beds had no significant effect on the duration of hospitalization. Fixed effects for the state of residence of the individual was used in the model with Rajasthan, Uttar Pradesh, and Gujarat were the only three state showing significant results.

**Table 7:** Tobit Regression Results for the Effect of Poor People Health Insurance Program on the Duration of Hospitalization

Duration of Hospitalization	Coefficient	95% CI	***P*** value
Public Health Insurance for the Poor			
Not enrolled (Reference)			
Enrolled	0.44	−0.47 - 1.35	0.346
Social Group			
Other Backward Classes (Reference)			
Scheduled Tribes	−1.20	−2.21 – 0.20	0.019
Scheduled Castes	−0.08	−1.07 – 0.90	0.870
Others	−0.56	−1.72 – 0.60	0.344
Chronic Illness			
No Chronic illness (Reference)			
Chronic Illness	3.15	1.96–4.33	< 0.001
Household Type			
Self-employed (Reference)			
Regular wage/Salary earning	0.38	−0.72 - 1.48	0.497
Casual labor	0.45	−0.34 - 1.26	0.263
Others	−0.03	−2.02 - 1.92	0.970
Age Groups			
0 to 18 years (Reference)			
19 to 40 years	−0.90	−1.87 - 0.05	0.065
41 to 60 years	1.08	−0.09 - 2.25	0.072
61 to 80 years	0.36	−1.14 - 1.88	0.631
Older than 80 years	0.44	−3.45 - 4.33	0.825
Household Size			
Small household (Reference)			
Medium household (5 to 8 members)	−0.15	−0.99 - 0.68	0.723
Large household (9 & more members)	−0.98	−2.22 - 0.26	0.124
Number of Hospital Beds in States			
Less than 10,000 beds (Reference)			
10,000 to 20,000 beds	0.38	−7.86 - 8.64	0.927
Greater than 20,000 beds	4.28	−3.69 - 12.26	0.292
Constant	3.35	−4.47 - 11.18	0.401

Results of the tobit regression model on the effects of poor people health insurance program on inpatient out-of-pocket health expenditures are shown in Table 8. Enrollment under the poor people health insurance program did not have any effect on inpatient OOP health expenditures. Duration of stay in hospital, graduate level education, age groups of 19 to 60 years, using a private hospital for treatment, admission in paying ward (general and special), and having ailments such as cancers, blood, endocrine, metabolic, eye, ear diseases, cardiovascular, respiratory diseases, skin, musculoskeletal, psychiatric, neurological diseases, and injuries had significant positive effect on the OOP health expenditures experienced by the individual. Utilization of AYUSH type of treatment had significant negative effect on OOP health expenditures compared to individuals using allopathic treatment. Factors such as location, social group, household type, household size, and number of hospital beds in states had no statistically significant effect on OOP health expenditures. Gujarat and Kerala states show significantly lower OOP expenses, keeping all other factors contact, than other states of India in the state fixed effects model.

**Table 8:** Tobit Regression Results for the Effect of Poor People Health Insurance Program on Inpatient Out-of-Pocket Health Expenditures

Out-of-Pocket Health Expenditures	Coefficient	95% CI	***P*** value
Public Health Insurance for the Poor			
Not enrolled (Reference)			
Enrolled	−950.36	− 2501.5 – 600.8	0.230
Duration of Stay in Hospital	521.40	435.3–607.5	0.000
Social Group			
Other Backward Classes (Reference)			
Scheduled Tribes	− 1073.94	− 2818.9 – 671.0	0.228
Scheduled Castes	−664.54	− 2328.9 – 999.8	0.434
Others	− 273.32	− 2251.1 – 1704.4	0.786
Education			
Illiterate (Reference)			
Primary/middle school educated	1104.02	−232.8 - 2440.8	0.105
Secondary school educated	285.39	− 2359.5 - 2930.3	0.832
Higher secondary school educated	− 1972.92	− 5096.8 - 1151.0	0.216
Diploma/graduate/post graduate educated	7634.86	2798.5–12,471.3	0.002
Household Type			
Self-employed (Reference)			
Regular wage/Salary earning	1034.10	−903.7 - 2971.9	0.295
Casual labor	− 1275.76	− 2654.2 - 102.6	0.070 0.934
Others	140.24	− 3201.5 – 3482.0	
Age Groups			
0–18 years (Reference)			
19 to 40 years	1857.13	−68.3 - 3782.6	0.059
41 to 60 years	2231.96	234.3–4229.6	0.029
61 to 80 years	87.75	− 2479.5 - 2655.0	0.947
Older than 80 years	− 1018.33	− 7587.8 - 5551.1	0.761
Household Size			
Small household (Reference)			
Medium household (5 to 8 members)	352.09	− 1064.2 - 1768.3	0.626
Large household (9 & more members)	2008.08	−79.56 - 4095.7	0.059
Number of Hospital Beds in States			
Less than 10,000 beds (Reference)			
10,000 to 20,000 beds	5850.75	− 7936.7 - 19,638.2	0.405
Greater than 20,000 beds	7440.12	− 5846.1 - 20,726.3	0.272
Nature of Treatment			
Allopathic treatment (Reference)			
AYUSH	− 9020.48	−16,224.0 - -1817.0	0.014
Level of Care Inpatient			
Sub-center/PHC/CHC (Reference)			
Public Hospital	949.24	− 958.0 - 2856.5	0.329
Private Hospital	3772.82	1004.0–6541.6	0.008
Type of Ward			
Free (Reference)			
Paying General	9095.49	6978.9–11,212.1	0.000
Paying Special	13,642.31	9856.4–17,428.3	0.000
Sector			
Rural (Reference)			
Urban	− 309.89	− 1754.5 - 1134.7	0.674
Nature of Ailment/ diseases			
Infections (Reference)			
Cancers, blood, endocrine, metabolic, eye, ear	3012.40	538.7–5486.1	0.017
Cardiovascular, respiratory diseases	3741.79	1137.1–6346.5	0.005
Gastrointestinal disease	− 1184.58	− 3790.0 -1420.8	0.373
Skin, musculoskeletal, psychiatric, neurological	2798.06	381.2–5214.9	0.023
Genitourinary, obstetric & childbirth	21.09	− 1858.7 - 1900.9	0.982
Injuries	4338.32	1727.1–6949.5	0.001
Constant	− 5660.85	−18,905.2 - 7583.5	0.402

## Discussions

Our study showed that poor people enrolled in the health insurance programs for the poor have higher incidence of hospitalization, but health insurance enrolment had no effect on the duration of hospitalizations. In general, health insurance coverage increases health care utilization because of higher access to care and changes in utilization behavior of both the insured and the health care provider. The results of our study are consistent with findings from other cross-sectional studies in Tamil Nadu and Maharashtra [27] which found significantly higher utilization of health care among the insured compared to the uninsured. Evidence from the US also indicates 61% reduction in inpatient hospital admissions and 40% reduction in emergency department visits among the uninsured compared to the insured population [11]. Lack of health insurance coverage usually forces people to delay or postpone medical care even when the medical care needed is of emergency type. With health insurance coverage, people can access health care with potentially lower financial risk. Currently, the health insurance for the poor people in India covers only inpatient services, which encourages patients to visit hospitals and get hospitalized instead of using basic primary health care services. Also, it creates a financial incentive for the provider to admit poor patients in the hospitals. Studies on hospitalization trends in India showed that annual hospitalization rate has increased from 16.6 to 37.0 per 1000 population from 1995 to 2014 [13]. Health insurance coverage affects total OOP expenses through two separate mechanisms – lower OOP expenses per unit of service and increased utilization of health services. Therefore, insurance coverage may or may not improve financial risk protection depending upon the degree of out-of-pocket price reduction of services and the change in the utilization levels [12].

Our study shows that incidence of chronic illnesses increases both the probability and the duration of hospitalizations. The findings are consistent with other results in the literature which show chronic diseases are important determinants of hospitalizations [43]. Since the health insurance programs for the poor do not cover outpatient services, people do not get preventive services or outpatient treatment for their illnesses during the initial stages of the diseases to better manage the disease progression and development of more severe chronic conditions. Although, public primary health care facilities provide free outpatient and preventive services, many poor individuals still face significant access barriers. In India, only 37% of the population in the rural areas have access to health care services within a 5-km radius and only 68% of the population have access to a basic outpatient health facility [44]. Further, India is facing demographic transition with increasing proportion of population in the higher age groups and epidemiological transition with increasing burden of non-communicable and chronic diseases [45]. In this study, we find that the incidence of hospitalization among poor people tends to increase with age. Elderly people over 80 years of age showed the highest incidence of hospitalization. These findings are consistent with another study in India that showed age of the individual as an important predictor of hospitalization [46]. Hospital readmissions [47] and increase in the number of comorbidities in an individual also increase with age [48]. Women in the age group of 19 to 40 years have higher incidence of hospitalization, which is an expected result for women in the reproductive age group [49, 50].

Our results show that medium and larger households have lower probability of hospitalization compared to smaller households. The odds of hospitalization for medium households is 0.77 and for the large households is 0.48. One of the probable reasons may be that larger households can arrange someone within the family to act as a caregiver in the case of illness or disability. This family caregiving may prevent hospitalization for many common conditions. It is also possible that larger household sizes are more averse to the likelihood of incurring high OOP expenses associated with hospitalizations. Evidence from US have shown that home health provision has reduced both the number of visits and duration of stay in the hospital [51]. Another reason may be related to the design of the health insurance program itself. Poor people health insurance programs in India cover hospitalization costs only for a limited number of household members. For example, health insurance programs such as RSBY and VAS in Karnataka are limited to a maximum of five members in the household, but some of the state health insurance programs in Andhra Pradesh and Tamil Nadu cover the whole family [14, 16, 17]. The RSBY program has a benefit ceiling of INR 30,000 and some of the state health insurance programs have much higher coverage limits, e.g., up to INR 200,000 in Andhra Pradesh [14]. These enrolment restrictions and relatively low maximum threshold adversely affect the households with higher number of members reducing their healthcare utilization and hospitalization.

People belonging to the scheduled tribe social group category had significantly lower duration of hospitalization compared to the other backward classes (reference group). Scheduled tribes have poor access to healthcare facilities since they usually live in areas with limited access to health care facilities [52]. This may explain their lower duration of hospitalizations. People belonging to the other disadvantaged groups including the backward classes and scheduled classes live in the cities or villages that are not as inaccessible as the tribal areas where the scheduled tribal people live. Thus, the access to the healthcare facilities and coverage by health insurance programs were significantly better for the other disadvantaged groups than the tribal population.

Our study showed that coverage under the public health insurance programs for the poor had no significant effect on OOP health expenditures for inpatient care. This is contradictory to the studies done in Andhra Pradesh [17, 20] and Karnataka [26] where significant reductions in OOP hospital expenditure were found with enrollment in the health insurance programs. However, other studies in Tamil Nadu and Andhra Pradesh [24] showed that households with health insurance coverage had higher OOP health expenditures. At the national level, another study by Karan et al. (2017) showed that the likelihood of incurring OOP health expenditures increased by 30% due to RSBY program and that RSBY has not been effective in reducing the burden of OOP health expenditures for poor households [16]. Despite the higher OOP expenses, the wellbeing of the poor improved due to the program. The evidence on OOP expenses is also mixed internationally with studies from Indonesia and Laos showing reductions in OOP health expenses for insurance coverage [53] but Vietnam study did not find any effect of health insurance program on OOP health expenditures [54]. We find that the OOP health expenditures increases with higher duration of hospital stay. A report from the World Bank in India [55] and a study based on low and middle income countries [56] showed that OOP expenses increases with increasing hospitalizations.

India has a pluralistic system of medical culture with a number of different types of alternative medical systems (apart from the allopathic systems of medicine) widely practiced and used [57]. The alternative systems of medicine (AYUSH) training programs are officially regulated by the government of India but there are many practicing healers in the country who have no formal training or qualifications. In our study, we found that the individuals who reported using AYUSH for their treatment incurred a lower OOP health expenditures compared to others who did not use AYUSH. This finding is not consistent with the results found for Tanzania [49] and Sri Lanka [58] where the utilization of traditional systems increased the OOP health expenditures. The reason may be that in India, the people who use AYUSH may be poorer and/or use it for medical conditions perceived to be not serious. In general, individuals with relatively complex medical conditions are more likely to use the modern or allopathic systems of medicine.

Our results showed that individuals who were admitted to private tertiary hospitals incurred higher OOP health expenditures compared to individuals admitted to public hospitals or primary health centers. A systematic review assessing OOP health expenditures across a number of countries found that the use of private healthcare facilities and inpatient admissions in private sector hospitals were associated with higher OOP health expenditures [59]. Evidence from Thailand also indicates higher OOP health expenditures for utilizing private hospitals [60]. The use of private sector hospitals for specific health services such as maternal health [61, 62], chronic disease treatment [63] were also associated with higher OOP expenditures. The level of hospital care (i.e., primary, secondary, and tertiary care), as expected, also affect OOP costs with higher expenses at higher levels and the likelihood of expenditures being catastrophic becomes significantly higher for tertiary hospitals [63]. People who are getting admitted to a paying ward incur higher OOP expenses compared to those who are admitted to a free ward. Most of the public health facilities in India provide inpatient admission free or at a very subsidized cost. Poor patients who are admitted in the paying wards incur higher OOP costs because the health insurance coverage is quite limited and patients may associate free bed with poor quality. India has a wide network of unregulated private sector hospitals with around 49% of total available hospitals being in the private sector [64].

In India, compared to the OOP expenses associated with infectious diseases, all other conditions and diseases showed significantly higher OOP inpatient health expenditures. India is facing an epidemiological transition from infectious diseases to chronic and non-communicable diseases [65]. The higher incidence and duration of hospitalizations for chronic diseases appear to be associated with higher OOP costs. These results are consistent with studies from India and other countries that found positive association between OOP expenses and incidence of medical conditions like disabilities, injuries due to road traffic accidents, and chronic illnesses [60, 66–71].

Poor people with a diploma/graduate/post graduate level of education showed higher OOP health expenditures compared to poor people who were illiterate. The results are consistent with the evidence from China which indicates that better educated individuals had higher OOP health expenditures [70]. Also, educational attainment had an effect on OOP costs for specific services. Studies in India [72] and Brazil [73] show that educated mothers reported higher OOP health expenditures. Our analysis shows that individuals in the age group 41 to 60 years had higher OOP health expenditures compared to those below 18 years. The odds of experiencing chronic diseases increase with age and chronic diseases are important determinants of hospitalizations and OOP costs. A number of studies showed that healthcare expenditures were significantly associated with age, and the effect of age on health expenditures was highest among the elderly [66, 74–79]. In India, however, the highest age group (81 years or over) did not show the highest OOP expenses. It appears that society assigns more value to the health and wellbeing of individuals in the age groups 41 to 60 years and 19–40 years compared to other age groups. India does not have any specific health insurance or social security program for the elderly population and provision of such specialized programs will help improve the wellbeing of the elderly.

### Limitations

The main limitations of this study arise from the use of secondary data. The contents and questions asked in the survey are not what an assessment of a program would have done to explore the specific research questions of this study. One of the most important concern is the lack of information on the coverage of public health insurance for the poor. The NSSO dataset includes a variable that indicates insurance coverage by all public health insurance schemes, i.e., all those covered by the government sponsored health insurance programs. Government sponsored health insurance schemes are many in India and includes Employee’s State Insurance Scheme (ESIS), Central Government Health Scheme (CGHS), and the poor people’s health insurance programs such as RSBY and other state health insurance programs. Clearly, government sponsored health insurance programs cover poor as well as non-poor households. Employees of the central and state governments are covered by government insurance and none of them are likely to be below the poverty line. It is also possible that many households covered by the insurance for the poor are not below the poverty line. Since the enrollment into the insurance for the poor happens infrequently, economic status of households may change from enrollment date to the date of the survey.

This research needed to identify the individuals and households who are covered by the government sponsored insurance for the poor. To identify the group covered by public insurance for the poor, a number of implicit assumptions were made: first, it is assumed that no insurance schemes of the government, other than the insurance program designed for the poor, covers the households or individuals below the poverty lines defined by the states. This conjecture is likely to be valid because governmental salary structure is such that almost no one covered by government employee health insurance program should be below the poverty line, irrespective of the size of the household. Second assumption is that all the poor individuals with insurance coverage must be enrolled in the public health insurance programs for the poor such as RSBY, RACHI etc.

These assumptions do not identify all the households and individuals covered under the government insurance schemes for the poor but identifies only those who are covered by the insurance scheme and are below the poverty line. The households that are below poverty line and not enrolled in the government sponsored health insurance programs are assumed to be the control group, i.e., the households that are eligible for participation in the health insurance program but were not enrolled. Poverty is a dynamic event where people move in and out of poverty and it is almost impossible for any program to be as dynamic as the underlying dynamics of social mobility. The households who were covered by the insurance for the poor at the time of the survey but were not below the poverty line can happen for two very different reasons. The first reason could be simple mis-targeting, i.e., the household should not be in the program based on the economic status of the household but were enrolled in the program. The second reason could be that the household belonged to the poverty category when the household got enrolled but the household graduated from poverty to above the poverty line during the intervening period. Since enrollment in the program and disenrollment from the program happens only infrequently, a certain percent of enrollees will be above the poverty line. Given the data we have, it is not possible to identify households who were covered by the insurance for the poor even though they were not poor.

In the empirical analysis, we have used the poverty line for 2014 to identify the individuals who were poor in 2014. Thus, our study focuses on the group who was below the poverty line and enrolled in any government health insurance program. Since the government health insurance scheme that covers individuals below the poverty line are the insurance schemes for the poor, it is likely that all those who are poor and covered by government health insurance are actually covered by the public health insurance for the poor. The implication of these implicit assumptions is that the study cannot conduct an assessment or evaluation of the insurance program for the poor. It is only assessing the differences in utilization and out-of-pocket expenses between the poor households and individuals covered by the public health insurance schemes for the poor and those not covered by the scheme. Therefore, it is not an assessment of the insurance program.

The cross-sectional nature of the data also creates another important limitation – the study can only observe the associations between health insurance coverage and other outcomes and no causal relationships can be determined. Thus, to understand the causal effect of the insurance programs on various outcomes, we need data over a number of years. Another limitation of the study is that the survey did not collect data from the floating population (people without any normal residence), but households residing in open spaces, roadside shelters and people who reside in the same place were listed. People residing in the protected residential areas of military, paramilitary, police areas and people in orphanages, rescue homes, etc., were not covered. The NSSO health survey data does not collect detailed consumption expenditure and the consumption expenditure in the NSSO survey does not differentiate between food and non-food expenditures. It should also be noted that all information is reported by the surveyed individuals in the households and some information required quite long recall time. Therefore, the data is prone to strategic, recall and other types of biases.

## Conclusions

The first set of analysis of this study examined the hospital utilization pattern by health insurance status of poor individuals. There are two aspects of hospital utilization – incidence of hospitalization and duration of hospitalization. The incidence indicates the need and/or willingness to get admitted in a hospital. Decision to become hospitalized is often not made by the patients; in most cases, individuals follow the instructions of physicians and other health care providers. Some individuals, however, may decide not to seek care from hospitals due to other barriers faced even though the hospitalization may be considered medically necessary. Once the patients decide to get admitted in hospitals, the length of stay is most likely determined by the health care providers and hospital managers.

The empirical results imply that the poor individuals enrolled in health insurance program are more likely to get admitted in a hospital than those who are not covered by health insurance. Incidence of hospitalization is a reflection of access to inpatient hospital services and it is not surprising to find that having insurance increases the likelihood of hospitalization. Even though the regression models do not show causal relationship, in this case it probably indicates causal pathway. Enrollment in insurance happens before utilization of hospital services and there exists no mechanism of obtaining insurance coverage due to the need for hospitalization. Therefore, only reasonable implication of the result would be that having insurance for inpatient services increases the incidence of hospitalization among poor individuals in India.

The second aspect of hospital service use is the intensity of service utilization after the patients are admitted. The empirical model indicates that insurance status had no relationship on the level of utilization of hospital services, measured by the length of stay. Since the insurance status had no effect on duration of hospital stay, health care providers did not discriminate between insured and uninsured once they are admitted in the hospitals. Again, this is not surprising for a number of reasons. The coverage limits in the health insurance programs for the poor is low and the coverage limits did not create any incentive for increasing the duration of hospitalizations by the physician. The other reason may be that physicians are driven by the intrinsic motivation to provide better care for the patients, irrespective of their health insurance coverage or their capacity to pay. There is always the possibility that the clinicians are unaware of the insurance status of the patient, which are usually handled by the administrative divisions of the hospitals, and thus their clinical decisions are independent of any health insurance enrolment status.

Apart from the insurance status, a number of other factors affect hospitalization and hospital duration. Chronic illnesses increase both the incidence and duration of hospitalization. Early detection by preventive screenings and early treatment initiation will help in decreasing disease progression, and thus reduce preventable hospitalizations. This early detection and treatment initiation could be delivered through the PHC system. India has a wide network of PHCs and the PHCs should be upgraded adequately with diagnostic and treatment facilities to detect and treat chronic conditions to help reduce hospital rates, the duration of hospitalizations, and the associated higher OOP healthcare costs for inpatient care. Many chronic diseases can be treated effectively in the ambulatory setting. Thus, better approaches to manage the chronic diseases in the outpatient settings should be considered for improving effective utilization of scarce medical resources.

The study found lower incidence of hospitalization among the larger size households. The insurance for the poor may not cover all individuals in the household and in many states, enrollment is limited to only five members of the household and these five members must be selected at enrollment in the program. Therefore, for large households, a number of members may not be covered by the program even though the household is enrolled in the insurance plan. Lack of insurance coverage for some members may reduce access and service utilization. Since the non-covered members do not get reimbursed for hospital expenses, they may end up showing lower rates of hospitalizations. This barrier in hospital utilization may adversely affect the health status of patients and overall health status of members in larger households. Thus, removing these enrolment restrictions will be helpful in improving hospital utilization for members of the larger households.

Our study shows that the Scheduled tribes in India have lower duration of hospitalization. Scheduled tribes have been traditionally neglected in the country with lower capacity to pay because of their limited employment opportunities in the formal sector, lack of access to cash, and their area of residence which is mostly located in the hilly and remote tribal areas of India. They also have poor physical access to health care facilities [52]. In addition, the enrolment of tribal people in the health insurance programs for the poor is quite low because of the presence of access barriers as well as general mistrust of any governmental programs by the tribal groups. One important policy implication is that all efforts should be undertaken to reduce access barriers for the Scheduled tribes. Government should initiate outreach program to reach this hard-to-reach group so that their enrolment in the insurance program can be expanded.

Both men and women in the age group 40 years or more had higher incidence of hospitalizations than other groups. Women in the age groups of 19 to 40 years have higher incidence of hospitalizations but men in the age group do not show higher incidence. The main reason for this may be that women in the reproductive age group have higher hospital admissions related to childbirth in healthcare facilities. In order to encourage safe deliveries, the Government of India promotes institutional deliveries through the Janani Suraksha Yojana (JSY) program, a conditional cash transfer scheme, which may have further increased hospitalizations among women in the reproductive age group.

Utilization of private hospitals have higher OOP health expenditures. Utilization of private hospitals, by itself, is not a problem but when the poor households obtain care from private hospitals, out-of-pocket expenses become too high for the poor households to afford. The poor households need to be protected from the high OOP health expenditures when they have no option other than using private hospitals due to non-availability of public facilities in the locality.

The private healthcare system in India is highly unregulated and patients often have no idea about the health care charges of hospitals. Government can regulate the private sector by fixing prices for different diagnosis groups so that households would become fully aware of total hospital bill for their specific medical conditions. Making the charges of hospitals more transparent will help protect households from the uncertainty related to hospital expenses. Government hospitals are potentially an important source of healthcare in India, especially for the poor. Unfortunately, many poor people do not use the government healthcare facilities because of their perceived low quality, poor infrastructure, absences of health care providers and significant travel distances. Strengthening the government health facilities will reduce access barriers for the poor and will help lower the OOP expenditures.

This research finds that specific diseases such as cancers, cardiovascular, endocrine, respiratory, neurological, obstetric and childbirth, and injuries have higher OOP inpatient health expenditures. Specific national health programs can be established to improve access to outpatient and inpatient care for some of these medical conditions. India is currently establishing a national health program for non-communicable diseases which is being piloted in some districts. Faster nation-wide implementation of this program will help the poor individuals to get specific health service packages. Also, the health insurance maximum benefit limits may be increased for the poor individuals who are suffering from these diseases. Increasing coverage limits for specific medical conditions may encourage “up coding” without a rigorous monitoring system and health information system should be strengthened to identify potential mis-classification of cases to increase reimbursement received by hospitals.

This study has helped identify the groups most affected by OOP inpatient expenses and should be useful to help design national insurance programs to protect health and wellbeing of the poor households. This investigation will serve as a basis for assessing India’s policy options to reduce financial burden due to OOP inpatient expenditures by households below the poverty line.

## Data Availability

The datasets used for the current study is available from the corresponding author on reasonable request. The data can also be obtained from the Ministry of Statistics and Implementation of the Government of India with appropriate permission.

## Abbreviations

OOP: Out-of-Pocket
UHC: Universal Health Coverage
SDGs: Sustainable Development Goals
GOI: Government of India
RSBY: Rashtriya Swasthiya Bima Yojana
RACHI: Rajiv Aarogyasri; Community Health Insurance
VAS: Vajpayee Aarogyasri Scheme
BPL: Below-Poverty-Line
NSSO: National Sample Survey Organization
DID: Difference-in-Difference
ESIS: Employee’s; State Insurance Scheme
CGHS: Central Government Health Scheme
AYUSH: Ayurveda, Yoga, Unani, Siddha and Homeopathy
INR: Indian Rupee
PHC: Primary Health Center
JSY: Janani Suraksha Yojana
